# Pharmacological Analysis of the Anti-epileptic Mechanisms of Fenfluramine in *scn1a* Mutant Zebrafish

**DOI:** 10.3389/fphar.2017.00191

**Published:** 2017-04-06

**Authors:** Jo Sourbron, Ilse Smolders, Peter de Witte, Lieven Lagae

**Affiliations:** ^1^Laboratory for Molecular Biodiscovery, Department of Pharmaceutical and Pharmacological Sciences, KU LeuvenLeuven, Belgium; ^2^Research group Experimental Pharmacology; ^3^Department of Development and Regeneration, Section Pediatric Neurology, University Hospital KU LeuvenLeuven, Belgium

**Keywords:** epilepsy, Dravet syndrome, Zebrafish, pharmacological modulation, GABA, glutamate, monoamines, sigma

## Abstract

Dravet syndrome (DS) is a genetic encephalopathy that is characterized by severe seizures and prominent co-morbidities (e.g., physical, intellectual disabilities). More than 85% of the DS patients carry an *SCN1A* mutation (sodium channel, voltage gated, type I alpha subunit). Although numerous anti-epileptic drugs have entered the market since 1990, these drugs often fail to adequately control seizures in DS patients. Nonetheless, current clinical data shows significant seizure reduction in DS patients treated with the serotonergic (5-hydroxytryptamine, 5-HT) drug fenfluramine (FA). Recent preclinical research confirmed the anti-epileptiform activity of FA in homozygous *scn1a* mutant zebrafish larvae that mimic DS well. Here we explored the anti-epileptiform mechanisms of FA by investigating whether selective agonists/antagonists of specific receptor subtypes were able to counteract the FA-induced inhibition of seizures and abnormal brain discharges observed in the *scn1a* mutants. We show that antagonists of 5-HT_1D_ and 5-HT_2C_ receptor subtypes were able to do so (LY 310762 and SB 242084, respectively), but notably, a 5-HT_2A_-antagonist (ketanserin) was not. In addition, exploring further the mechanism of action of FA beyond its serotonergic profile, we found that the anti-epileptiform brain activity of FA was significantly abolished when it was administered in combination with a σ_1_-agonist (PRE 084). Our study therefore provides the first evidence of an involvement of the σ_1_ receptor in the mechanism of FA. We further show that the level of some neurotransmitters [i.e., dopamine and noradrenaline (NAD)] in head homogenates was altered after FA treatment, whereas γ-aminobutyric acid (GABA) and glutamate levels were not. Of interest, NAD-decreasing drugs have been employed successfully in the treatment of neurological diseases; including epilepsy and this effect could contribute to the therapeutic effect of the compound. In summary, we hypothesize that the anti-epileptiform activity of FA not only originates from its 5-HT_1D_- and 5-HT_2C_-agonism, but likely also from its ability to block σ_1_ receptors. These findings will help in better understanding the pharmacological profile of compounds that is critical for their applicability in the treatment of DS and possibly also other drug-resistant epilepsies.

## Introduction

Epilepsy is a severe neurological disease affecting 65 million people worldwide (Moshé et al., 2015). It is marked by abnormal electrical activity in the brain and is associated with precipitous recurrent episodes of involuntary movements and/or loss of consciousness. Various epilepsy syndromes have been described with different etiologies (e.g., genetic, structural, metabolic, infectious or unknown cause). In general, multiple genes and/or environmental factors are implicated in the pathogenesis of epilepsy but also monogenic epilepsy syndromes exist (Williams and Battaglia, 2013). Of interest, these monogenic pathologies can be mimicked rather easily by genetic animal models (Dinday and Baraban, 2015; Sourbron et al., 2016).

A *de novo* mutation in the *SCN1A* gene (sodium channel, voltage gated, type I alpha subunit) has been proven to be the cause in more than 85% of the DS patients (Mastrangelo and Leuzzi, 2012). This monogenic epilepsy syndrome, which was also known as severe myoclonic epilepsy of infancy (SMEI), is an uncommon but severe encephalopathy that starts in the first year of life (Dravet, 2011). Moreover it is one of the most drug-resistant epilepsy syndromes, highlighting the need for new innovative drugs with novel mechanisms of action.

Even though new AEDs have been developed over the past two decades, the treatment of drug-resistant epilepsy has not improved significantly (Ventola, 2014). Notably, some interesting progress has been made in the understanding of the mechanisms underlying epilepsy, revealing novel targets for future AEDs (Löscher et al., 2013). For instance, current evidence underscores the role of SER (5-HT) in epilepsy and it has been suggested that serotonergic drugs have great potential in the treatment of various epilepsy conditions (Gharedaghi et al., 2014).

One interesting example of a successful epilepsy therapy based on serotonergic modulation relates to the use of fenfluramine (FA) as an add-on drug in a small clinical trial of DS patients (Ceulemans et al., 2012). FA is a potent SER releaser and also directly acts on multiple 5-HT receptor subtypes, 14 of which have been described in humans. Prospective data also demonstrated the efficacy and safety of FA in treating DS patients (Schoonjans et al., 2015), which led to the initiation of clinical Phase III studies early in 2016^1^.

Zebrafish (*Danio rerio*) are increasingly used in neurological research (Best and Alderton, 2008) and the high genetic homology between ZF and human (ca. 70%) makes them an attractive model to study genetic diseases (Howe et al., 2013). More than 80% of the known epilepsy-related genes are found in the ZF genome and different groups were able to generate ZF epilepsy models by specific gene manipulation, recapitulating the main characteristics of the human disease, e.g., fever-sensitive myoclonic epilepsy (*CHD2* and *SCN1A*) (Suls et al., 2013; Zhang et al., 2015) and fever-associated epilepsy (*STX1B*) (Schubert et al., 2014). In addition, the phenotype of homozygous *scn1Lab* mutant ZF larvae (herein referred to as *scn1a* mutant larvae) has been studied and mirrors the clinical features of DS well. Significantly, these mutants did not respond to currently available AEDs and only valproic acid and FA were effective in treating seizures, as seen in DS patients (Dinday and Baraban, 2015; Sourbron et al., 2016).

Our pharmacological investigations using *scn1a* mutant larvae showed that stimulation of the 5-HT_2B_ receptor (by BW 723C86, a selective 5-HT_2B_-agonist) did not generate an anti-epileptiform effect whereas the application of selective 5-HT_1D_- (GR 46611), 5-HT_2A_- (TCB 2) or 5-HT_2C_- (lorcaserin) agonists resulted in a significant decrease of epileptiform locomotor and brain activity (Sourbron et al., 2016). However, direct evidence that these 5-HT receptor subtypes are implicated in the pharmacological activity of FA is presently lacking. Moreover, *in vitro* assays interrogating potential targets suggested beta_2_-adrenergic- (β_2_) and sigma- (σ) antagonism as possibly involved in the mechanism of FA (Parthena et al., 2016). These receptors have relatively high protein similarity compared to their human counterparts, which amounts to 68.2 and 79.5 % for the β_2_ receptor and σ_1_ receptor, respectively [as calculated by EMBOSS stretcher, (Rice et al., 2000)]. In addition, they show a pronounced expression in the ZF brain (comparable to the human proteins) (Wang et al., 2009; Moritz et al., 2015). Structural data regarding the σ_2_ receptor subtype are more ill-defined, even though selective compounds with high affinity for the binding site are available (Rhoades et al., 2014).

In this study, the mechanistic profile of FA was explored in *scn1a* mutant larvae by using subtype selective agonists and antagonists of serotonergic, beta_2_-adrenergic- and sigma receptors. We demonstrate that the anti-epileptiform activity of FA originates both from its agonistic action at 5-HT_1D_ and 5-HT_2C_ receptors and its antagonistic action at σ_1_ receptors. Moreover, we found a significant decrease of certain monoamines in the head homogenates of ZF larvae after FA treatment. The results obtained allowed us to put forward a mechanistic hypothesis regarding the clinical efficacy of FA in the treatment of DS.

## Materials and Methods

### Compounds and Their Maximum Tolerable Concentration (MTC)

Compounds were purchased from Tocris Bioscience, except for the 5-HT_2A_-antagonist, ketanserin (Sigma-Aldrich) and (±)FA that was a gift from Prof. Berten Ceulemans (Child Neurology, University Hospital Antwerp, Belgium). Agonists and antagonists were selected by their high and selective affinity for a specific receptor (**Tables 1, 2**). In addition, all compounds have a cLogP value higher than one (cLogP > 1.0) ensuring a good bioavailability in ZF larvae (Milan, 2003; García-Pedraza et al., 2013; Watry and Lu, 2013) (**Tables 1, 2**). These LogP values were calculated by an interactive calculator after conversion of the chemical names to SMILES (cLogP range between 1.21 and 4.59). Compounds were dissolved in DMSO (99.9% spectroscopy grade, Acros Organics) (stock solution), and immediately before use diluted in embryo medium to achieve a final DMSO concentration of 0.1% w/v that also served as VHC control.

**Table 1:** Compounds with selective agonistic or antagonistic activity at the beta_2_-adrenergic (β_2_) or the sigma receptors (σ_1_ and σ_2_).

Receptor	Full name	MTC (μM)	MW (g/mol)	cLogP	K_i_ (nM)	K_i_ (other receptor subtypes) (nM)	Reference
**AGONIST**							
σ_1_-Agonist	PRE 084	6.25	317.43	3.63	2.2	13091.0 (σ_2_)	Nakazato et al., 1999
**ANTAGONIST**							
β_2_-Antagonist	ICI 118551	50.00	277.41	2.93	1.2	120.0 (β1)	Wenzel et al., 2009
						257.0 (β3)	
σ_1_-Antagonist	NE 100	6.25	327.47	4.35	1.0	212.0 (σ_2_)	Nakazato et al., 1999
σ_2_-Antagonist	SM 21	6.25	453.92	4.20	145.0	1050.0 (σ_1_)	Matsumoto et al., 2007

**Table 2:** Compounds with selective agonistic or antagonistic activity at specific SER (5-HT) receptor subtypes.

5-HT receptor subtype	Full name	MTC (μM)	MW (g/mol)	cLogP	K_i_ (nM)	K_i_ (other 5-HT receptor subtype) (nM)	Reference
**AGONIST**							
1D-Agonist	PNU 109291	25.00	409.52	2.99	0.9	1700.0 (2A)	Choi et al., 2008
						1090.0 (1A)	
2A-Agonist	NBOH-2C-CN	1.25	312.37	2.96	1.3	132.0 (2C)	Hansen et al., 2014
2C-Agonist	Ro 600175	1.25	226.68	1.21	1.0	31.6 (2A)	Tian et al., 2015
						3981.1 (1A)	
**ANTAGONIST**							
1D-Antagonist	LY 310762	2.50	435.44	4.44	249.0	–	García-Pedraza et al., 2013
2A-Antagonist	Ketanserin	0.63	372.89	3.86	0.8	21.6 (2C)	Watry and Lu, 2013
						28.7 (2A)	
2B-Antagonist	SB 204741	6.25	467.78	4.59	1.0	100.0 (2B)	Watry and Lu, 2013
						158.0 (2A)	
2C-Antagonist	SB 242084	0.31	365.86	2.37	12.0	–	Watry and Lu, 2013

Before performing pharmacological experiments, the MTC of each compound was determined. Six dpf WT *scn1Lab*^+/+^ ZF larvae (hereinafter referred to as WT larvae) were incubated in a 96-well plate, one larva per well (tissue culture plate, flat bottom, FALCON^®^, USA), with different concentrations of compound or VHC at 28°C on a 14/10 h light/dark cycle (medium was replenished daily). Each larva was checked under the microscope during a period of 48 h for the following signs of toxicity: decreased or no touch response upon a light touch of the tail, loss of posture, body abnormalities, edema, anomalies in heart rate or circulation and death. The MTC was determined using 12 WT ZF larvae (6 dpf) and defined as the highest concentration at which none of these larvae showed signs of toxicity within 48 h of exposure. Compounds were used at their MTC (**Tables 1, 2**), whereas FA was used at a final concentration of 25 μM as described before (Sourbron et al., 2016).

### Zebrafish Research

#### Zebrafish Maintenance and Experimental Set-up

ZF (*Danio rerio*) heterozygous for the *scn1Lab* mutation (*scn1Lab*^+/-^), backcrossed with *Tupfel longfin* WT (WT *scn1Lab*^+/+^), were a generous gift of Dr. H. Baier (Freiburg, Germany). The adult ZF were kept at 28.0°C, on a 14/10 h light/dark cycle under standard aquaculture conditions. Fertilized eggs were collected via natural spawning. Genotyping of these ZF adults and embryos was performed as described previously (Sourbron et al., 2016). The embryos and larvae were kept in petridishes in embryo medium containing 1.5 mM HEPES, pH 7.6, 17.4 mM NaCl, 0.21 mM KCl, 0.12 mM MgSO_4_ and 0.18 mM Ca(NO_3_)_2_ in an incubator at 28.0°C, likewise on a 14/10 h light/dark cycle. *Scn1a* mutant larvae were selected by their darker appearance, lack of a swim bladder and slight curvature of the body (Schoonheim et al., 2010).

All ZF experiments were approved by the Ethics Committee of the University of Leuven (Ethische Commissie van de KU Leuven, approval number 154/2015) and by the Belgian Federal Department of Public Health, Food Safety & Environment (Federale Overheidsdienst Volksgezondheid, Veiligheid van de Voedselketen en Leefmileu, approval number LA1210199).

#### Locomotor Behavior

*Scn1a* mutant larvae and WT larvae were arrayed in a 96-well plate (one larva per well) and treated at 6 dpf with VHC, compound or co-treatment of compounds for a 22 h period as performed previously (Sourbron et al., 2016). The co-treatment of FA and subtype selective 5-HT-antagonists and/or the σ_1_-agonist was performed to investigate the mechanism of action of FA. After incubation at 28°C on a 14/10 h light/dark cycle and 30 min chamber habituation 7 dpf larvae were placed in an automated tracking device (ZebraBox^TM^ apparatus; Viewpoint, Lyon, France) and their locomotor behavior was evaluated during 10 min under dark conditions, as described before (Sourbron et al., 2016). Locomotor activity was quantified by the lardist parameter (total distance in large movements) and plotted in cm (ZebraLab^TM^ software, Viewpoint, Lyon, France). Data were collected and pooled together from two or three independent experiments with at least eight larvae per treatment condition. The locomotor data were analyzed by normalizing the locomotor activity of treated ZF larvae against age-matched VHC-treated larvae (method A), and by calculating the percentage of *scn1a* mutant larvae with epileptiform activity below or above a 70% threshold value using the cumulative locomotor activity during 10 min of each larva separately (method B). Method A has been described previously (Sourbron et al., 2016) and is performed for both *scn1a* mutant larvae and WT larvae. The threshold value used in method B is based on sensitivity analyses with different cut-off values in ‘10%-steps’; i.e., 10, 20, 30, 40, 50, 60, 70, 80, and 90% of the average cumulative locomotor activity during 10 min of VHC-treated *scn1a* mutant larvae per experiment (data available in Supplementary Tables S1–S5).

#### Forebrain Local Field Potential Recordings

The epileptiform brain activity was measured by non-invasive surface recordings from the skin above the forebrain (Zdebik et al., 2013). *Scn1a* mutant and WT larvae were treated on 6 dpf as described above [see Locomotor Behavior (Single and Combined)] and recordings were performed at 7 dpf. ZF larvae were immobilized by 2% low-melting-point agarose (Invitrogen) (no paralytic was used).

The recording electrodes were made of borosilicate glass with microfilament (GC150TF-7.5, Harvard Apparatus, UK), pulled with DMZ Universal Puller (Zeitz, Germany) to an opening 15–20 microns, and filled with aCSF made from: 124 mM NaCl, 2 mM KCl, 2 mM MgSO_4_, 2 mM CaCl_2_, 1.25 mM KH_2_PO_4_, 26 mM NaHCO_3_ and 10 mM glucose. The differential signal was amplified 10,000 times using DAGAN 2400 amplifier (Minneapolis, MN, USA), band pass filtered at 0.3–300.0 Hz and digitized at 2.0 kHz via a PCI-6251 interface (National Instruments, UK) using WindEDR (John Dempster, University of Strathclyde, UK). Single recordings were performed for 10 min and epileptiform activity was quantified according to the duration of spiking paroxysms as described before (Sourbron et al., 2016). Electrograms were analyzed with Clampfit 10.2 software (Molecular devices corporation, USA). Spontaneous epileptiform activity was defined as events in which the amplitude exceeded three times the baseline and lasted longer than 50 ms. At least 10 (up to 50) ZF larvae were used per experimental condition.

#### Neurotransmitter Determination

The heads of 7 dpf ZF larvae were used to quantify the amount of the neurotransmitters 5-HT, NAD, DOP, GABA and GLUT. Both *scn1a* mutant larvae and WT larvae were treated with either VHC or FA (*n* = 48 for both treatment arms, leading to a total of 192 larvae). Six heads per tube were homogenized and data acquisition was carried out as described before (Sourbron et al., 2016). Thereafter, the amount of neurotransmitter (in nmol) was determined and expressed as nmol neurotransmitter per mass head homogenate (mg).

### Statistical Analysis

GraphPad Prism 5 software (GraphPad Software, Inc.) was used for statistical analyses.

The locomotor activity was analyzed by the methods described above [see Locomotor Behavior (Single and Combined)]. The results obtained with method A were analyzed by One-way ANOVA and subsequent Dunnett’s multiple comparison tests. The results obtained with method B were performed for the individual experiments by contingency tables, followed by Fisher’s exact tests.

Electrographic brain activity was analyzed by a Student’s *t*-test [if data passed the normality test (D’Agostino & Pearson omnibus normality test)]. Mann–Whitney *U* tests were used if the data did not pass the normality test, as described before (Sourbron et al., 2016). To determine if the genotype (*scn1a* mutant vs. WT) and/or FA treatment affected the neurotransmitter content, data were analyzed by a two-way ANOVA for genotype and FA treatment as the inter-subject factors. Regarding the analyses of single vs. triple experiments [whether or not combined with FA; see Anti-epileptiform Activity Induced by Sigma_1_-Antagonism and 5-HT_1D&2C_-Agonism (Triple Treatment) and Counteraction of FA’s Anti-epileptiform Activity by Sigma1-Agonism and 5-HT_1D&2C_-Agonism (Triple Treatment), respectively] Mann–Whitney *U* tests were applied because the data did not pass the normality test.

For all analyses, differences between a treatment group and the equivalent control groups were considered statistically significant if the *p*-value was below 0.05 (*p* < 0.05).

## Results

**FIGURE 1:**
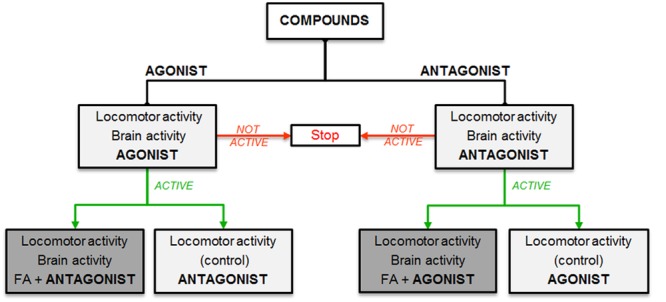
**Experimental workflow.** Selection of compounds based on hypothesized mechanisms of FA: stimulation (agonist, left side) or inhibition (antagonist, right side) of specific receptors. Light gray boxes show single treatment and dark gray boxes represent combinatorial treatment with FA.

To explore the anti-epileptiform mechanism(s) of FA, we used an experimental workflow that is depicted in **Figure 1**.

First, we selected compounds with specific agonistic or antagonistic activity at distinct receptor subtypes (i.e., 5-HT_1D_, 5-HT_2A_, 5-HT_2C_ receptors, and β_2_, σ_1_, σ_2_ receptors, respectively) and studied their effects on the locomotor and brain activity of *scn1a* mutant larvae.

The locomotor data were analyzed by normalizing the locomotor activity of treated ZF larvae against age-matched VHC-treated larvae (method A), and by calculating the percentage of *scn1a* mutant larvae with epileptiform activity below or above a 70% threshold value (method B). Method A was used to evaluate the results of both *scn1a* mutant larvae and WT larvae and has the advantage to demonstrate non-specific effects, e.g., a decrease in both mutant and WT larvae by sedative effects. Method B was applied to the data of the *scn1a* mutant larvae and has the advantage to account for the inherent variability of locomotor assays (i.e., different larvae at different days) since each larva is analyzed separately for each individual experiment.

In case of a significant decrease of epileptiform activity for a specific agonist/antagonist [as shown by at least one method of locomotor analysis (A and/or B) and confirmed by forebrain LFP recordings], we continued by treating larvae in separate experiments with a combination of FA and a compound (combinatorial treatment) with a counteractive profile of the agonist/antagonist previously used (in other words: in case of an active agonist, we combined FA with an antagonist and vice versa). Thereafter, we also tested the activity of the agonist/antagonist used in the combinatorial treatment as a single compound (control experiment). Finally, we performed triple treatment experiments to examine additional effects when combining all anti-epileptiform compounds. Similarly, we explored FA’s pharmacological profile by combining FA with triple treatment of compounds with the counteractive profile of the agonists/antagonists previously used.

### Anti-epileptiform Activity Induced by Sigma_1_-Antagonism, 5-HT_1D&2C_-Agonism

**FIGURE 2:**
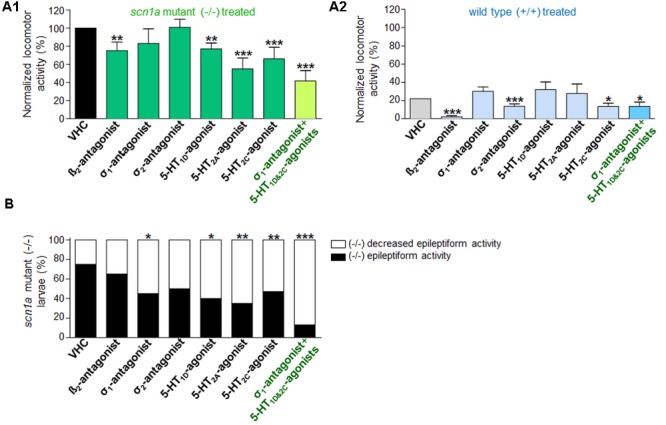
**Activity profile of the selective beta_2_-antagonist (β_2_), sigma_1_-antagonist (σ_1_), sigma_2_-antagonist (σ_2_), subtype selective 5-HT-agonists (1D, 2A, 2C) and triple treatment with anti-epileptiform compounds [σ_1_-antagonist and subtype selective 5-HT-agonists (1D and 2C)] (locomotor behavioral assays).** Locomotor activity was normalized against VHC-treated *scn1a* mutant larvae and displayed as a percentage ± SD. **(A1)** Normalized locomotor activity of treated *scn1a* mutant larvae (-/-). **(A2)** Normalized locomotor activity of treated WT larvae (+/+). **(B)** Percentage of *scn1a* mutant larvae with epileptiform activity below (white area) or above the threshold value (black area). Treatment with the selective β_2_-antagonist, σ_1_-antagonist, the subtype selective 5-HT-agonists (1D, 2A, 2C) and triple treatment with anti-epileptiform compounds [σ_1_-antagonist and subtype selective 5-HT-agonists (1D and 2C)] induced anti-epileptiform locomotor activity. A statistical difference is indicated by: ^∗^*p* < 0.05, ^∗∗^*p* < 0.01 and ^∗∗∗^*p* < 0.001 vs. VHC-treated controls. *n* = 16–30 ZF larvae for all experimental conditions.

**FIGURE 3:**
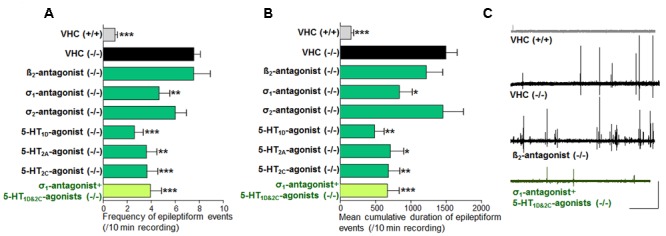
**Activity profile of the selective beta_2_-antagonist (β_2_), sigma_1_-antagonist (σ_1_), sigma_2_-antagonist (σ_2_), subtype selective 5-HT-agonists (1D, 2A, 2C) and triple treatment with anti-epileptiform compounds [σ_1_-antagonist and subtype selective 5-HT-agonists (1D and 2C)] (forebrain LFP recordings).** Quantification of brain recordings of VHC-treated WT larvae [VHC(+/+), *n* = 42], VHC-treated *scn1a* mutant larvae [VHC(-/-), *n* = 41] and compound-treated *scn1a* mutant larvae [compound(-/-)]. **(A)** Frequency of epileptiform events during 10 min recording. **(B)** Mean cumulative duration of epileptiform events during 10 min recording. Treatment with the selective β_2_-antagonist (*n* = 19) or the σ_2_-antagonist (*n* = 14) did not affect epileptiform brain activity in *scn1a* mutant larvae. In contrast, the σ_1_-antagonist (*n* = 20), 5-HT_1D_-agonist (*n* = 10), 5-HT_2A_-agonist (*n* = 10), the 5-HT_2C_-agonist (*n* = 14) and triple treatment with anti-epileptiform compounds [σ_1_-antagonist and subtype selective 5-HT-agonists (1D and 2C); *n* = 11] significantly reduced the frequency and the mean cumulative duration of epileptiform brain activity in *scn1a* mutant larvae. A statistical difference is indicated by: ^∗^*p* < 0.05, ^∗∗^*p* < 0.01 and ^∗∗∗^*p* < 0.001 vs. VHC-treated *scn1a* mutant larvae. **(C)** Visualization of representative electrograms of 7 dpf ZF larvae: VHC-treated WT larva [VHC (+/+)], VHC-treated *scn1a* mutant larva [VHC (-/-)]; β_2_-antagonist-treated *scn1a* mutant larva [β_2_-antagonist (-/-)]; triple treatment of *scn1a* mutant larva with anti-epileptiform compounds [σ_1_-antagonist+5-HT_1D&2C_-agonists (-/-)]; scale bars: 1 mV, 30 s.

Blocking the β_2_-adrenergic receptor (by ICI 118551) or the σ_1_ receptor (by NE 100) resulted in a significant decrease of epileptiform locomotor activity of the *scn1a* mutant larvae, as identified by one of the two methods assessing locomotor activity (i.e., method A and method B, respectively) (**Figures 2A,B**). The β_2_-adrenergic receptor antagonist, however, also reduced the locomotor activity of WT larvae, whereas the σ_1_-antagonist did not (**Figure 2A1**). Subsequently, treated *scn1a* mutant larvae were subjected to brain activity recordings, showing that only the σ_1_-antagonist (NE 100) was able to significantly reduce epileptiform brain activity (**Figures 3A,B**). Representative traces of brain activity during 5 min recordings are depicted in **Figure 3C**. σ_2_-antagonism (by SM 21) did not decrease epileptiform locomotor or brain activity in *scn1a* mutant larvae but lowered the locomotor activity of WT larvae (**Figures 2, 3**).

Previous work showed evidence for a role of 5-HT_1D_, 5-HT_2A_, and 5-HT_2C_ receptors in seizure modulation (Sourbron et al., 2016). Here we reconfirmed the anti-epileptiform effect of a distinct set of 5-HT-agonists (**Figures 2A,B** and **Table 1**). Agonism at the 5-HT_1D_ (by PNU 109291) and 5-HT_2A_ receptor (by NBOH-2C-CN) also decreased epileptiform brain activity of *scn1a* mutant larvae (**Figures 3A,B**) and did not affect the locomotor activity of WT larvae (**Figure 2A1**). Similarly, the 5-HT_2C_-agonist (Ro 600175) decreased locomotor activity of *scn1a* mutant but also of the WT larvae (**Figure 2A1**). However, consecutive brain recordings proved the selective anti-epileptiform activity of this compound in *scn1a* mutant larvae (**Figures 3A,B**).

### Anti-epileptiform Activity of FA and the Counteraction by Sigma_1_-Agonism or 5-HT_1D&2C_-Antagonism (Combinatorial Treatment)

**FIGURE 4:**
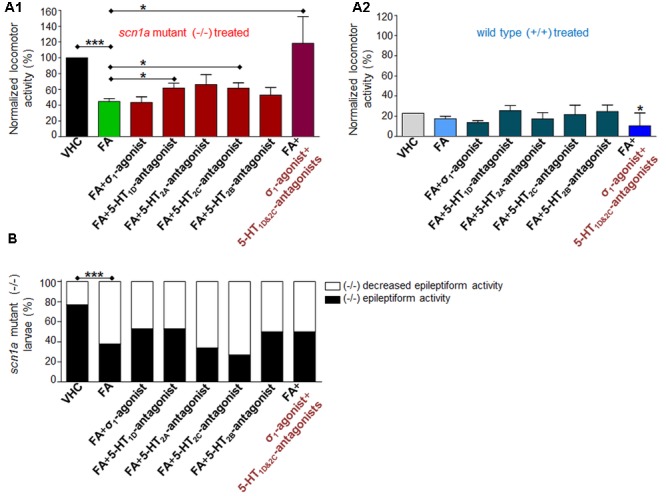
**Activity profile of FA [single treatment with FA and combinatorial treatment of FA with the sigma_1_-agonist (σ_1_) or subtype selective 5-HT-antagonists (1D, 2A, 2C, 2B) or triple treatment of epileptiform compounds [σ_1_-agonist and subtype selective 5-HT-antagonists (1D and 2C)] (locomotor behavioral assays)].** Locomotor activity was normalized against VHC-treated *scn1a* mutant larvae (-/-) and displayed as a percentage ± SD. **(A1)** Normalized locomotor activity of treated *scn1a* mutant larvae (-/-). **(A2)** Normalized locomotor activity of treated WT larvae (+/+). **(B)** Percentage of *scn1a* mutant larvae with epileptiform activity below (white area) or above the threshold value (black area). Single treatment with FA induced anti-epileptiform locomotor activity (FA), which was counteracted by combinatorial treatment with the 5-HT_1D_-antagonist or the 5-HT_2C_-antagonist or triple treatment of epileptiform compounds [σ_1_-agonist and subtype selective 5-HT-antagonists (1D and 2C)]. A statistical difference is indicated by: ^∗^*p* < 0.05 and ^∗∗∗^*p* < 0.001 vs. VHC-treated controls (or FA-treated larvae). *n* = 16–30 ZF larvae for all experimental conditions.

**FIGURE 5:**
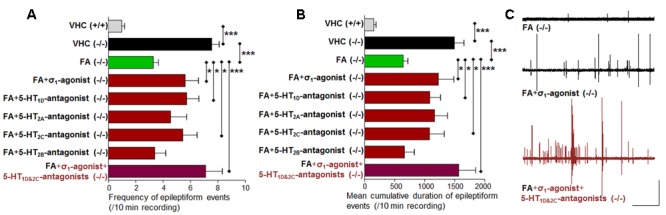
**Activity profile of FA [single treatment with FA and combinatorial treatment of FA with the sigma_1_-agonist (σ_1_) or subtype selective 5-HT-antagonists (1D, 2A, 2C, 2B) or triple treatment of epileptiform compounds [σ_1_-agonist and subtype selective 5-HT-antagonists (1D and 2C)] (forebrain LFP recordings)].** Quantification of brain recordings of VHC-treated WT larvae [VHC(+/+), *n* = 42], VHC-treated *scn1a* mutant larvae [VHC(-/-), *n* = 41] and compound-treated *scn1a* mutant larvae [compound(-/-)]. **(A)** Frequency of epileptiform events during 10 minutes recording. **(B)** Mean cumulative duration of epileptiform events during 10 min recording. Single treatment with FA reduced epileptiform brain activity (*n* = 50), which was counteracted by combinatorial treatment with the σ_1_-agonist (*n* = 13), the 5-HT_1D_-antagonist (*n* = 18) or the 5-HT_2C_-antagonist (*n* = 16). A statistical difference is indicated by ^∗^*p* < 0.05 and ^∗∗∗^*p* < 0.001. **(C)** Visualization of representative electrograms of 7 dpf ZF larvae: FA-treated *scn1a* mutant larva [FA(-/-)]; FA-treated *scn1a* mutant larva in combination with the σ_1_-agonist [FA+ σ_1_-agonist(-/-)] or triple treatment with epileptiform compounds [σ_1_-agonist+5-HT_1D&2C_-antagonists (-/-)]; scale bars: 1 mV, 30 s.

In order to explore the mechanisms of action of FA, we combined FA with the σ_1_-agonist (PRE 084) or 5-HT subtype selective antagonists (**Figure 1** and **Table 2**). First, we reconfirmed the anti-epileptiform activity of FA in both assays (locomotor and brain recordings) (**Figures 4, 5**) as shown before (Dinday and Baraban, 2015; Sourbron et al., 2016). The σ_1_-agonist was unable to counteract the anti-epileptiform activity of FA in the locomotor assay (**Figures 4A,B**), although brain recordings revealed a statistically significant counteraction (**Figures 5A,B**). Treatment with the 5-HT_1D_-antagonist (LY 310762) or the 5-HT_2C_-antagonist (SB 242084) significantly counteracted the decrease in locomotor activity as elicited by FA in *scn1a* mutant larvae (**Figures 4A,B**). This was not the case for the 5-HT_2A_-antagonist (ketanserin). The locomotor behavior was not significantly altered in age-matched WT larvae by combinatorial treatments (**Figure 4A2**), pointing to a selective effect on *scn1a* mutant larvae. Interestingly, quantification of the corresponding electrograms confirmed the significant counteraction of the 5-HT_1D_-antagonist and 5-HT_2C_-antagonist (**Figures 5A,B**). Representative traces of brain activity during 5 min recording are depicted in **Figure 5C**. Finally, we performed experiments with a selective 5-HT_2B_-antagonist (SB 204741, negative control). This antagonist was unable to counteract the anti-epileptiform activity of FA in the locomotor (**Figures 4A,B**) and the brain recording assays (**Figures 5A,B**).

### No Anti-epileptiform Activity of Sigma_1_-Agonism or 5-HT-Antagonism

**FIGURE 6:**
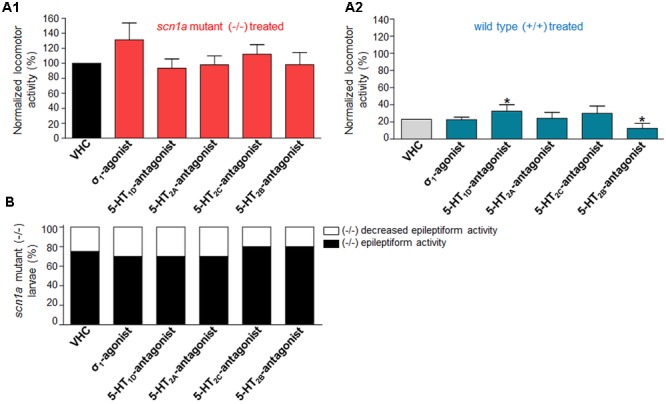
**Activity profile of the sigma_1_-agonist (σ_1_) and subtype selective 5-HT-antagonists (1D, 2A, 2C, 2B) (locomotor behavioral assays).** Locomotor activity was normalized against VHC-treated *scn1a* mutant larvae (-/-) and displayed as a percentage ± SD. **(A1)** Normalized locomotor activity of treated *scn1a* mutant larvae (-/-). **(A2)** Normalized locomotor activity of treated WT larvae (+/+). **(B)** Percentage of *scn1a* mutant larvae with epileptiform activity below (white area) or above the threshold value (black area). Single treatment with the σ_1_-agonist (σ_1_) and the subtype selective 5-HT-antagonists (1D, 2A, 2C, 2B) did not significantly affect the epileptiform locomotor behavior. A statistical difference is indicated by: ^∗^*p* < 0.05 vs. VHC-treated controls. *n* = 20 ZF larvae for all experimental conditions.

Subsequently, we performed single treatment experiments to investigate whether the compounds, used in combinatorial treatment with FA [see Anti-epileptiform Activity of FA and the Counteraction by Sigma_1_-Agonism or 5-HT_1D&2C_-Antagonism (Combinatorial Treatment)], affected the locomotor activity of ZF larvae in the absence of FA (**Figure 6**). Treatment with the σ_1_-agonist did not significantly influence the locomotor activity of ZF larvae, although a slight statistically insignificant increase in the epileptiform behavior of *scn1a* mutant larvae was present (*p* > 0.05, **Figure 6A**). The pharmacological inhibition of the 5-HT_1D_, 5-HT_2A_, and the 5-HT_2C_ receptor did not alter epileptiform locomotor activity in *scn1a* mutant larvae (**Figures 6A,B**). The 5-HT_1D_-antagonist and the 5-HT_2B_-antagonist significantly affected locomotor activity of age-matched WT larvae (**Figure 6A1**).

### Anti-epileptiform Activity Induced by Sigma_1_-Antagonism and 5-HT_1D&2C_-Agonism (Triple Treatment)

Single treatment (σ_1_-antagonist, 5-HT_1D_- or 5-HT_2C_-agonist) decreased the abnormal (epileptiform) locomotor activity (range 17–34% decrease compared to VHC; **Figure 2A1**). Triple treatment (combination of the aforementioned three compounds) decreased the epileptiform locomotor activity to a greater extent (58% decrease compared to VHC, **Figure 2A1**), which is similar to the decrease elicited by FA (>55% decrease compared to VHC; **Figure 4A1**). Subsequent brain recordings confirmed these anti-epileptiform effects, though no major differences were observed between single and triple treatment (**Figures 3, 5**). Consistently, statistical analyses did not show any significant differences between triple treatment and single treatments (*p* < 0.05).

### Counteraction of FA’s Anti-epileptiform Activity by Sigma_1_-Agonism and 5-HT_1D&2C_-Antagonism (Triple Treatment)

Combinatorial treatment of FA with single treatment of the σ_1_-agonist, 5-HT_1D_- or 5-HT_2C_-antagonist counteracted FA’s decrease in locomotor activity (range 0–41% increase relative to FA; **Figure 4A1**). Triple treatment (combination of the aforementioned three compounds) counteracted FA’s decrease in locomotor activity more profoundly (89% increase relative to FA; **Figure 4A1**). Consistently, brain recordings confirmed the counteraction of FA’s anti-epileptiform effect and showed that triple treatment counteracted FA’s effect more profoundly (116–144% increase relative to FA for the frequency and cumulative duration of epileptiform events, respectively; **Figure 5**), in comparison to the single treatment counteraction experiments (74–91% relative to FA for the frequency and cumulative duration of epileptiform events, respectively; **Figure 5**). Overall, these more pronounced counteractive effects by triple treatment compared to single treatments were not statistically significant (*p* > 0.05).

### Neurotransmitter Changes after FA Treatment

**FIGURE 7:**
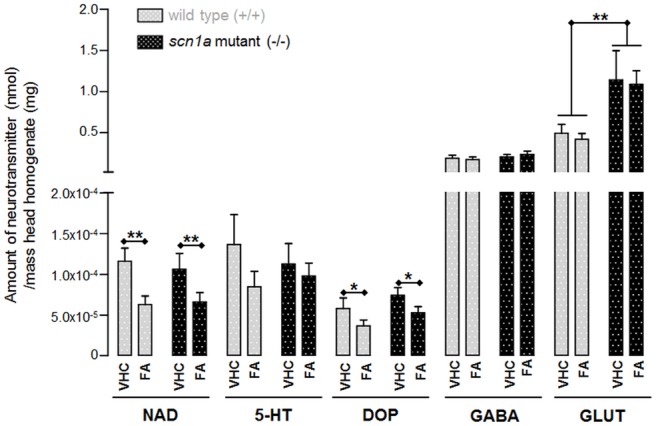
**Neurotransmitter content in head homogenates of ZF larvae (nmol/mg).** Data for each condition were collected from eight samples with each six heads (*n* = 48). ^∗^*p* < 0.05 and ^∗∗^*p* < 0.01 indicate a statistically significant alteration of some neurotransmitters (Supplementary Table S6; Two-way ANOVA).

Our data indicate an overall reduction of two out of three monoamines after FA treatment, i.e., DOP and NAD (**Figure 7**). The most pronounced and significant decrease was shown for NAD (**Figure 7** and Supplementary Table S6).

The only statistical significant difference between both genotypes was the GLUT content which was significantly higher in *scn1a* mutant larvae (-/-) when compared to WT larvae (+/+). However, GLUT content was not significantly altered after FA treatment (**Figure 7** and Supplementary Table S6).

## Discussion

Dravet syndrome is one of the most drug-resistant forms of epilepsy that typically starts in the first year of life (Nolan et al., 2011). Recently, it was shown that the serotonergic drug FA is a potent add-on AED in DS patients (Ceulemans et al., 2012). Although this compound shows great promise for future therapeutic regimens in the treatment of DS and possibly other drug-resistant epileptic syndromes, it is presently unknown which 5-HT receptor subtypes are involved in its action and whether other mechanisms beyond its serotonergic profile play a part. This situation hampered a full mechanistic appreciation of the clinical performance of the compound.

### Serotonergic Activity (5-HT)

In previous experimental work using *scn1a* mutant larvae, which mimic well the epileptiform features of DS in ZF, we have shown that selective pharmacological agonists of the 5-HT_1D_, 5-HT_2A_ and 5-HT_2C_ receptor subtypes (GR 46611, TCB 2 and lorcaserin, respectively) substantially decreased the epileptiform activity. Of importance, stimulation of other 5-HT receptor subtypes was not met with success. Using an identical approach here, we explored whether selective antagonists of 5-HT_1D_, 5-HT_2A_ and 5-HT_2C_ receptor subtypes were able to counteract the FA-induced inhibition of epileptiform locomotor and brain activity observed in *scn1a* mutant larvae. We show that antagonists of 5-HT_1D_ and the 5-HT_2C_ receptor subtypes were able to do so (LY 310762 and SB 242084, respectively), but notably, the 5-HT_2A_-antagonist (ketanserin) was not.

Of importance, and in line with our previous findings (Sourbron et al., 2016), a 5-HT_2B_-antagonist (SB 204741) did not counteract the anti-epileptiform activity of FA. Consequently, we can conclude that the 5-HT_2B_ receptor subtype, that is proven to cause cardiotoxic effects after prolonged use of FA (Rothman et al., 2000), is not involved in the anti-epileptiform effect of FA.

We also examined the anti-epileptiform activity of the subtype selective 5-HT_1D_-, 5-HT_2A_- and 5-HT_2C_-antagonists without combinatorial FA treatment (i.e., single treatment) as other studies have shown that blocking these receptor subtypes can protect against seizures in other seizure/epilepsy models. For example, the 5-HT_2A_-antagonist used in this study, ketanserin, protected against seizures induced by hippocampal kindling in cats (Wada et al., 1992). The same antagonist was also involved in increasing the latency to audiogenic seizures in DBA/2 mice (Semenova and Tiku, 1997). Consistently, the 5-HT_2C_-antagonist, mesulergine, inhibited myoclonic jerks induced by 5-HTP in guinea pigs. This latter antagonist primarily blocks the 5-HT_2C_ receptor and displays affinity for the 5-HT_2A_ receptor subtype (Pappert et al., 1998). Selective pharmacological blockade of the 5-HT_1D_ receptor also inhibited myoclonic jerks in the same model of generalized myoclonus (Hagan et al., 1995). However, our data clearly show that antagonists for these 5-HT receptor subtypes did not show any anti-epileptiform activity in our ZF model of DS.

### Beta_2_-Adrenergic Activity (β_2_)

The activity of FA has always been labeled as 5-HT related, as confirmed in this study. Nonetheless, recent *in vitro* data suggested antagonism of sigma (σ) and beta_2_-adrenergic (β_2_) receptors as part of its pharmacological spectrum (Parthena et al., 2016). Hence, in this study we used the *scn1a* mutant larvae to explore the anti-epileptiform activity of σ- and β_2_- antagonism in *in vivo* conditions.

A selective antagonist of the β_2_-adrenergic receptor (ICI 118551) was able to significantly reduce the locomotor activity of *scn1a* mutant larvae. This is in agreement with the findings of Luchowska et al. (2001), that showed an anti-epileptiform effect against maximal electroshock-induced seizures in mice by antagonizing the β-adrenergic receptor. Additionally, β-adrenergic receptors have been implicated in audiogenic seizures and pindolol, a potent β-antagonist, was found to attenuate audiogenic seizures in DBA/2 mice (Lints and Nyquist-Battie, 1985). Nevertheless, in this work we show that the percentage of *scn1a* mutant larvae with decreased epileptiform activity was not significantly changed. In addition, the locomotor activity of WT larvae was decreased, suggesting a non-specific alteration of locomotor activity (i.e., potential sedative or muscle-relaxant effect). In line with these latter findings, the β_2_-antagonist did not decrease epileptiform brain activity. Thus, our *in vivo* data imply that the β_2_-adrenergic receptor is not involved in modulating epileptiform activity in the ZF DS model.

### Sigma Activity (σ_1_ and σ_2_)

As far as the sigma receptors are concerned, the σ_1_-antagonist (NE 100) decreased both epileptiform behavior and especially brain activity in *scn1a* mutant larvae. On the other hand, the σ_1_-agonist (PRE 084) did not counteract FA’s decrease in abnormal locomotor activity (behavioral assays, **Figure 4**), whereas brain activity recordings clearly demonstrated a statistically significant counteraction (**Figure 5**). Locomotion is regulated in a complex way in different parts of the brain (Dunn et al., 2016) and it is possible that these neuronal activities are not always consistent with the brain activity alterations recorded by a single electrode positioned at the forebrain. Alternatively, it is also conceivable that the σ_1_-agonist (PRE 084) does not uniformly distribute over the brain, and that especially the peripheral areas probed by the electrode accumulated more compound resulting in a more pronounced effect.

Altogether, the data therefore suggest an involvement of the σ_1_ receptor in the mechanism of FA, beyond its serotonergic profile. In contrast, treatment with the σ_2_-antagonist (SM 21) did not reduce epileptiform activity in *scn1a* mutant larvae. This result was somewhat anticipated as most studies underline the role of the σ_2_ subtype receptor in cancer-related diseases, rather than in brain-related disorders (Rousseaux and Greene, 2015).

Of interest, a number of studies demonstrate the role of σ_1_ receptors in neurological diseases like epilepsy (Rousseaux and Greene, 2015) and overstimulation of σ_1_ receptors has been associated with GLUT-related excitatory effects (via NMDA receptors) (Borlot et al., 2014; Nicita et al., 2014; Rousseaux and Greene, 2015). In addition, blockade of σ_1_ receptors was associated directly with anti-epileptiform activity (Matsumoto et al., 2002). In contrast, also σ_1_-agonists have also been shown to exert clear anti-seizure effects (Meurs et al., 2007; Guo et al., 2015), and possibly σ_1_-agonist/antagonists might exert different activities depending on the seizure/epilepsy model. Anyhow, our data show that σ_1_-agonism was ineffective in reducing epileptiform events, whereas σ_1_-antagonism likely plays a role in the mechanism of FA in the ZF DS model.

### Neurotransmitter Content

The neurotransmitter content analysis of *scn1a* mutants compared to WT only demonstrated a statistically significant increase in GLUT (*p* = 0.0036). This finding is in line with the neuro-excitatory properties related to GLUT and its role in epilepsy (Meldrum, 1994). Hence it is not surprising that decreasing glutamatergic effects is one of the main targets for currently available AEDs (Landmark, 2007). Nonetheless, the anti-epileptic compound FA did not alter GLUT levels. Interestingly, the concentration of other neurotransmitters (i.e., NAD and DOP) in head homogenates was altered after FA treatment. Our data indicate a significant DOP decrease by almost one third compared to the VHC treated group (*p* = 0.0335). The NAD content was even nearly halved when comparing the FA vs. the VHC treated group (*p* = 0.0035). The latter result is therefore in line with previous work that demonstrated a FA-related decrease of the NAD content in rat brain (Calderini et al., 1975).

As 5-HT-agonism (Astorne Figari et al., 2014) but also σ-antagonism (Monnet et al., 1992) can result in decreased NAD signaling, we hypothesize that the reduction of NAD content after FA treatment as observed in this study is due the effects of FA at 5-HT_2C_ and σ_1_ receptors. Of interest, elevated NAD transmission has been associated with some cases of epilepsy (Fitzgerald, 2010) and although some studies show contradictory results (Svob Strac et al., 2016), also evidence exists supporting the use of NAD-decreasing drugs in the treatment of neurological diseases, including epilepsy (Fitzgerald, 2015).

Of importance, the concentrations measured encompass the total intra- and extracellular levels, so inter-synaptic level alterations were not assessed.

The following, yet incomplete, hypothesis emerges regarding the pharmacological profile of FA that underlies the treatment of DS and possibly other *SCN1A*-related epilepsies. Of importance, the present study used only one agonist or antagonist at their respective MTCs for the exploration of the involvement of some type of receptors, and further experiments using alternative methods (e.g., a genetic approach using receptor gene knock-down or overexpression) are needed to confirm the present results.

*SCN1A* mutations, as a major monogenic cause of DS, lead to a malfunction of the encoded voltage-gated sodium channels 1.1 (Na_v_1.1). In mammalian (rodent) DS models these channels are mainly affected in inhibitory (GABAergic) interneurons and to a lesser extent in excitatory pyramidal neurons (Dutton et al., 2013; Mistry et al., 2014). As a result, neuronal inhibition is impaired, which is assumed to cause seizures (Wong et al., 2016). Our data suggest that FA reduces epileptiform activity by stimulating 5-HT_2C_ and 5-HT_1D_ receptors, leading to enhanced GABAergic neurotransmission (as proven for 5-HT_2C_ receptors) (Shen and Andrade, 1998; Higgins et al., 2014), and by a σ_1_-antagonistic action that can reduce the excitatory neurotransmission by modulating NMDA responses (Rousseaux and Greene, 2015). In addition, FA also significantly decreases the NAD brain content that might contribute to its anti-epileptic effects.

## Conclusion

This work extends our current understanding of the anti-epileptic mechanisms of FA, beyond its serotonergic activity. Using an *in vivo scn1a* mutant ZF model we demonstrated that the anti-epileptiform activity of FA not only originates from its 5-HT_1D_- and 5-HT_2C_-agonism, but likely also from its ability to block σ_1_ receptors. These findings will help to understand the pharmacological profile of compounds that is critical for their applicability in the treatment of DS and possibly also other drug-resistant epilepsies.

## Author Contributions

All authors took part in the outlines of the research. JS carried out all the zebrafish experiments and data-analysis. Neurotransmitter analyses were done by IS. JS and PdW were involved in the manuscript and figure preparation. All authors approved the final manuscript version.

## Conflict of Interest Statement

LL obtains consultancy honoraria from Zogenix. No conflict of interest is declared regarding materials, methods or results in this research. The other authors declare that the research was conducted in the absence of any commercial or financial relationships that could be construed as a potential conflict of interest.

The other authors declare that the research was conducted in the absence of any commercial or financial relationships that could be construed as a potential conflict of interest.

## Footnotes

1 http://Zogenix.com

## Abbreviations

5-HT: 5-hydroxytryptamine (serotonin)
5-HTP: 5-Hydroxy tryptophan
aCSF: artificial cerebrospinal fluid
AED: anti-epileptic drug
cLogP: calculated LogP
DMSO: dimethylsulfoxide
DOP: dopamine
dpf: days post fertilization
DS: Dravet syndrome
EEG: electroencephalogram
GABA: γ-aminobutyric acid
GLUT: glutamate
lardist: distance in large movements (cm)
LFP: local field potential
ms: milliseconds
MTC: maximum tolerable concentration
mV: millivolt
NAD: noradrenaline
Na_v_1.1: sodium channel protein type 1 subunit alpha isoform 1
Na_v_1.5: sodium channel protein type 1 subunit alpha isoform 5
NMDA: *N*-methyl-D-aspartate
*SCN1A*: sodium channel, voltage gated, type I alpha subunit (human)
*scn1Lab*: sodium channel, voltage-gated, type I like, alpha b (zebrafish)
SER: serotonin
VHC: vehicle
WT: wild type
ZF: zebrafish
