# Supplementary material for: Pharmacological Analysis of the Anti-epileptic Mechanisms of Fenfluramine in scn1a Mutant Zebrafish
Source: Front Pharmacol. 2017 Apr 6;8:191. doi: 10.3389/fphar.2017.00191 (PMC5382218; doi:10.3389/fphar.2017.00191)
Supplement: Supplementary file 1 [file Table_1.doc]

Supplementary Material

Pharmacological analysis of the anti-epileptic mechanisms of fenfluramine in *scn1a* mutant zebrafish

**Jo Sourbron, Ilse Smolders, Peter de Witte^*^and Lieven Lagae**

*** Correspondence:** Peter de Witte; peter.dewitte@pharm.kuleuven.be

**Table S1:** Determination of the *threshold* value (“limit”) in VHC-treated *scn1Lab^-/-^* mutant larvae, above which a larva was considered as seizing. This was achieved by sensitivity analyses of potential *threshold* values: 90; 80; 70; 60; 50; 40; 30; 20 and 10%. The highest *threshold* value that still resulted in a statistical difference was 80%, although this was borderline significance (*p*=0.0484). By decreasing the *threshold* value, chances increase to incorrectly diminish significant differences between the studies groups. Hence, a *threshold* value of 70% appeared to be a relevant *threshold* value which moreover appears to be a good predictor of the activity in LFP recordings. A statistical difference is indicated by a **p*<0.05 vs VHC-treated controls. No statistical difference is left blank. *n*=20 ZF larvae for all experimental conditions.

| **Percentage calculated of average sum of 10 VHC-treated *scn1Lab^-/-^* mutant larvae during 600 s (threshold values)** | **90%** | **80%** | **70%** | **60%** | **50%** | **40%** | **30%** | **20%** | **10%** |
| --- | --- | --- | --- | --- | --- | --- | --- | --- | --- |
| 1^st^ experiment (6368.08) | 5731,272 | 5094,464 | **4457,656** | 3820,848 | 3184,04 | 2547,232 | 1910,424 | 1273,616 | 636,808 |
| 2^nd^experiment (4679.48) | 4211,532 | 3743,584 | **3275,636** | 2807,688 | 2339,74 | 1871,792 | 1403,844 | 935,896 | 467,948 |
| **Amount of treated *scn1Lab^-/-^* mutant larvae (1^st^ + 2^nd^ experiment)** |  |  |  |  |  |  |  |  |  |
| VHC-treated *scn1Lab^-/-^* mutant larvae below threshold value | 13 | 16 | **17** | 18 | 19 | 19 | 19 | 19 | 19 |
| VHC-treated *scn1Lab^-/-^* mutant larvae below threshold value | 7 | 4 | **3** | 2 | 1 | 1 | 1 | 1 | 1 |
| NE100-treated *scn1Lab^-/-^* mutant larvae above threshold value | 8 | 9 | **9** | 10 | 14 | 17 | 17 | 18 | 19 |
| NE100-treated *scn1Lab^-/-^* mutant larvae below threshold value | 12 | 11 | **11** | 10 | 6 | 3 | 3 | 2 | 1 |
| *p*-value Fisher's exact test | 0,2049 | 0,0484* | **0,0187*** | 0,0138* | 0,0915 | 0,605 | 0,605 | 1 | 1 |

**Table S2-S5:** Calculation of the *threshold* value (“limit”) in VHC-treated *scn1Lab^-/-^* mutant larvae, above which a larva was considered as seizing**.** This *threshold* value was defined as 70% of the average cumulative locomotor activity of 10 individual VHC-treated homozygous *scn1Lab^-/-^* mutants per experiment. These four tables represent two independent experiments (duplicate) where we demonstrate the higher amount of homozygous *scn1Lab^-/-^* mutants with decreased epileptiform activity (below the *threshold* value). This was consistently done for all locomotor experiments.

|  | **VHC-treated *scn1Lab^-/-^* mutant larvae (1^st^ experiment)** | | | | | | | | | | | | | | |
| --- | --- | --- | --- | --- | --- | --- | --- | --- | --- | --- | --- | --- | --- | --- | --- |
| *Time (s)* | **1** | | **2** | | **3** | | **4** | **5** | **6** | | **7** | **8** | | **9** | **10** |
| *0* | 0 | | 1061,2 | | 2631,6 | | 961,6 | 2521,8 | 0 | | 4 | 824,4 | | 289,6 | 1247,6 |
| *100* | 432 | | 1767 | | 1117 | | 1181,6 | 2 | 652,6 | | 1545,6 | 1744,4 | | 1019,6 | 1320,6 |
| *200* | 721 | | 419,4 | | 360,6 | | 1290,6 | 1603,8 | 0 | | 1136,6 | 545,2 | | 1447,2 | 1408,8 |
| *300* | 1382 | | 629 | | 1695,6 | | 1345,2 | 1456,2 | 812,4 | | 925 | 118 | | 542 | 1314,8 |
| *400* | 1531,4 | | 1555 | | 2 | | 1692,6 | 1984 | 962,4 | | 1136,4 | 1467,8 | | 1553 | 978,2 |
| *500* | 856,6 | | 1130,8 | | 976,4 | | 667,2 | 1756,6 | 1333,4 | | 582,4 | 1573 | | 916,2 | 1547,8 |
| **SUM** | **4923** | | **6562,4** | | **6783,2** | | **7138,8** | **9324,4** | **3760,8** | | **5330** | **6272,8** | | **5767,6** | **7817,8** |
| ***AVERAGE OF SUM*** | ***6368,08*** | | | ***Amount of zebrafish larvae above “limit”*** | | | | | | ***9*** | | |  | | |
| ***70% OF AVERAGE (“limit”)*** | ***4457,656*** | | | ***Amount of zebrafish larvae below “limit”*** | | | | | | ***1*** | | |  | | |
|  | | **NE 100-treated *scn1Lab^-/-^* mutant larvae (1^st^ experiment)** | | | | | | | | | | | | | |
| *Time (s)* | | 1 | 2 | | 3 | | 4 | 5 | 6 | | 7 | 8 | | 9 | 10 |
| *0* | | 182,4 | 0 | | 0 | | 162 | 1544,4 | 1103,2 | | 463,6 | 1240,6 | | 589,2 | 2076,4 |
| *100* | | 0 | 53,8 | | 0 | | 241,2 | 2 | 392,8 | | 0 | 11,6 | | 1147,2 | 1391 |
| *200* | | 0 | 0 | | 0 | | 145,8 | 0 | 1362 | | 59,6 | 1483,6 | | 1323,6 | 1382,6 |
| *300* | | 428 | 0 | | 1341 | | 359,8 | 650,4 | 1278,6 | | 651,6 | 1873,4 | | 659 | 1610,2 |
| *400* | | 984,8 | 0 | | 2 | | 68 | 0 | 1601,8 | | 207,6 | 2123,2 | | 1367,6 | 1935,2 |
| *500* | | 1059,8 | 310,2 | | 1339,4 | | 0 | 1109,8 | 1640 | | 272,8 | 882,2 | | 616,8 | 1497,8 |
| **SUM** | | **2655** | **364** | | **2682,4** | | **976,8** | **3306,6** | **7378,4** | | **1655,2** | **7614,6** | | **5703,4** | **9893,2** |
| ***Amount of zebrafish larvae above “limit”*** | | | | ***4*** | |  | | | | | | | | | |
| ***Amount of zebrafish larvae below “limit”*** | | | | ***6*** | |  | | | | | | | | | |
| **Table S2-S5:** **cont’d** | | | | | |  | | | | | | | | | |

|  | **VHC-treated *scn1Lab^-/-^* mutant larvae (2^nd^ experiment)** | | | | | | | | | | | | |
| --- | --- | --- | --- | --- | --- | --- | --- | --- | --- | --- | --- | --- | --- |
| *Time (s)* | **1** | **2** | | **3** | **4** | **5** | **6** | | **7** | **8** | | **9** | **10** |
| *0* | 11,2 | 766,8 | | 569,8 | 761,6 | 1154,6 | 1378 | | 0 | 811,2 | | 1205,6 | 779 |
| *100* | 648 | 745 | | 179,4 | 740,4 | 1046,2 | 343,2 | | 12,8 | 1021,8 | | 1258,6 | 1288 |
| *200* | 0 | 1004,2 | | 32,6 | 858,4 | 705,8 | 1063,4 | | 14,4 | 1092,8 | | 1185,4 | 142,2 |
| *300* | 1090,8 | 739 | | 0 | 1790 | 1604,8 | 1478 | | 0 | 526 | | 141 | 746,4 |
| *400* | 1233,8 | 1372 | | 1149,2 | 892 | 653,8 | 752,8 | | 350,2 | 885,6 | | 1064 | 1542,6 |
| *500* | 1045 | 933,2 | | 1240 | 117,6 | 1265,4 | 868 | | 0 | 1823,6 | | 669,6 | 0 |
| **SUM** | **4028,8** | **5560,2** | | **3171** | **5160** | **6430,6** | **5883,4** | | **377,4** | **6161** | | **5524,2** | **4498,2** |
| ***AVERAGE OF SUM*** | ***4679,48*** | | ***Amount of zebrafish larvae above “limit”*** | | | | | ***8*** | | |  | | |
| ***70% OF AVERAGE (“limit”)*** | ***3275,636*** | | ***Amount of zebrafish larvae below “limit”*** | | | | | ***2*** | | |  | | |

|  | **NE 100-treated *scn1Lab^-/-^* mutant larvae (2^nd^ experiment)** | | | | | | | | | | | |
| --- | --- | --- | --- | --- | --- | --- | --- | --- | --- | --- | --- | --- |
| *Time (s)* | 1 | 2 | | 3 | | 4 | 5 | 6 | 7 | 8 | 9 | 10 |
| *0* | 706 | 32,6 | | 13,6 | | 1597,4 | 101,2 | 700,4 | 216 | 404 | 970,6 | 1156,2 |
| *100* | 1418,6 | 201 | | 0 | | 2386,6 | 5,6 | 8,4 | 1109,2 | 0 | 1574,6 | 1740,8 |
| *200* | 532,4 | 229,6 | | 78,2 | | 1005,6 | 790,2 | 943,2 | 1410,2 | 19,4 | 1686,8 | 817 |
| *300* | 422,8 | 22 | | 1622 | | 491,8 | 378,8 | 589,8 | 1476,8 | 305,6 | 1493,4 | 704,8 |
| *400* | 843,4 | 727,6 | | 636,2 | | 1662,6 | 1053 | 453,2 | 1105,6 | 1539 | 1356,8 | 1529 |
| *500* | 1183 | 1021,4 | | 736,8 | | 1618,4 | 445,8 | 49,6 | 786,2 | 215 | 1151,8 | 1952 |
| **SUM** | **5106,2** | **2234,2** | | **3086,8** | | **8762,4** | **2774,6** | **2744,6** | **6104** | **2483** | **8234** | **7899,8** |
| ***Amount of zebrafish larvae above “limit”*** | | | ***5*** | |  | | | | | | | |
| ***Amount of zebrafish larvae below “limit”*** | | | ***5*** | |  | | | | | | | |

**Table S6:** Two-way ANOVA analyses for treatment and genotype as inter-subject factors regarding neurotransmitter content per mass head homogenate (nmol/mg). A statistical difference is represented in bold and indicated by a **p*<0.05 or ***p*<0.01. No statistical difference is left blank.

| Source of variation | ***F*** | ***p*** |
| --- | --- | --- |
| **NAD** |  |  |
| Interaction | 1.986x10^-1^ | 0.6588 |
| Genotype | 5.250x10^-2^ | 0.8202 |
| Treatment (FA) | 9.932x10^3^ | **0.0035**** |
| **5-HT** |  |  |
| Interaction | 5.436x10^-1^ | 0.4671 |
| Genotype | 4.608x10^-2^ | 0.8316 |
| Treatment (FA) | 1.709x10^3^ | 0.2018 |
| **DOP** |  |  |
| Interaction | 2.831x10^-4^ | 0.9867 |
| Genotype | 2.737x10^3^ | 0.1078 |
| Treatment (FA) | 4.935x10^3^ | **0.0335*** |
| **GABA** |  |  |
| Interaction | 9.979x10^-1^ | 0.3264 |
| Genotype | 1.062x10^3^ | 0.3117 |
| Treatment (FA) | 3.024x10^-1^ | 0.5867 |
| **GLUT** |  |  |
| Interaction | 4.226x10^-2^ | 0.8386 |
| Genotype | 1.011x10^1^ | **0.0036**** |
| Treatment (FA) | 1.849x10^-2^ | 0.8928 |

NAD=noradrenaline; 5-HT=serotonin; DOP=dopamine; GABA=ɣ-aminobutyric acid;
GLUT=glutamate; FA=fenfluramine
